# The Effect of Malrotation of Tibial Component of Total Knee Arthroplasty on Tibial Insert during High Flexion Using a Finite Element Analysis

**DOI:** 10.1155/2014/695028

**Published:** 2014-05-08

**Authors:** Kei Osano, Ryuji Nagamine, Mitsugu Todo, Makoto Kawasaki

**Affiliations:** ^1^Sugioka Memorial Hospital, Bone and Joint Center, 3-6-1 Kashiiteriha, Higashi-ku, Fukuoka 813-0017, Japan; ^2^Research Institute for Applied Mechanics, Kyushu University, 6-1 Kasuga-koen, Kasuga, Fukuoka 816-8580, Japan; ^3^School of Medicine, University of Occupational and Environmental Health, 1-1 Iseigaoka, Yahata-nishi-ku, Kitakyushu, Fukuoka 807-8555, Japan

## Abstract

One of the most common errors of total knee arthroplasty procedure is a malrotation of tibial component. The stress on tibial insert is closely related to polyethylene failure. The objective of this study is to analyze the effect of malrotation of tibial component for the stress on tibial insert during high flexion using a finite element analysis. We used Stryker NRG PS for analysis. Three different initial conditions of tibial component including normal, 15° internal malrotation, and 15° external malrotation were analyzed. The tibial insert made from ultra-high-molecular-weight polyethylene was assumed to be elastic-plastic while femoral and tibial metal components were assumed to be rigid. Four nonlinear springs attached to tibial component represented soft tissues around the knee. Vertical load was applied to femoral component which rotated from 0° to 135° while horizontal load along the anterior posterior axis was applied to tibial component during flexion. Maximum equivalent stresses on the surface were analyzed. Internal malrotation caused the highest stress which arose up to 160% of normal position. External malrotation also caused higher stress. Implanting prosthesis in correct position is important for reducing the risk of abnormal wear and failure.

## 1. Introduction

Total knee arthroplasty (TKA) has been a common surgical treatment for severe osteoarthritis of the knee. High knee flexion after TKA is of increasing concern for people who are younger and more physically active as well as people living in the Middle East and Asia where high flexion is culturally required for activities of daily living [1]. Advancement of TKA prostheses design contributed to the improvement of clinical performance including a widened range of motion and a prolonged prosthesis survivorship. However, surgical procedure as well as prosthesis design is very important for knee kinematics and long-term survivorship of prosthesis. One of the most common procedure errors is a malrotation of tibial component [2]. Although the ideal axial alignment of tibial component is still being discussed [3–6], it was reported that malrotation affects the distribution of the stress on contact surface of tibial insert [7, 8], which might cause the early failure of tibial insert [9], and, moreover, leads to knee pain after TKA [2].

In the literature, several studies analyzed the stress on polyethylene insert and contact areas using computational models [9–19]. Although some of them used finite element analysis (FEA) [10–14, 16–18], most of their analyses focused on walking gait or static analysis [10, 14, 15]. A few studies used dynamic models to analyze the stress distribution [12, 16–18]. Our group developed simplified three-dimensional FEA models to reproduce the implanted knee kinematics and investigated the stress distribution on the tibial insert [16, 17]. The objective of this study is, therefore, to analyze the effect of malrotation of tibial component on tibial insert during squatting maneuver using our FEA model.

## 2. Methods

**Figure 1:**
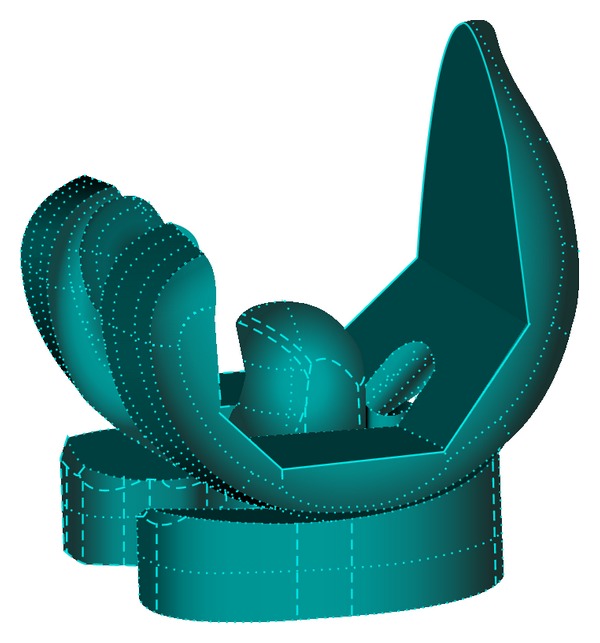
Prosthesis used in the current analysis. Femoral and tibial components are assumed to be rigid and UHMWPE tibial insert was assumed to be elastic-plastic. Two pegs of femoral component were removed for simplification.

**Figure 2:**
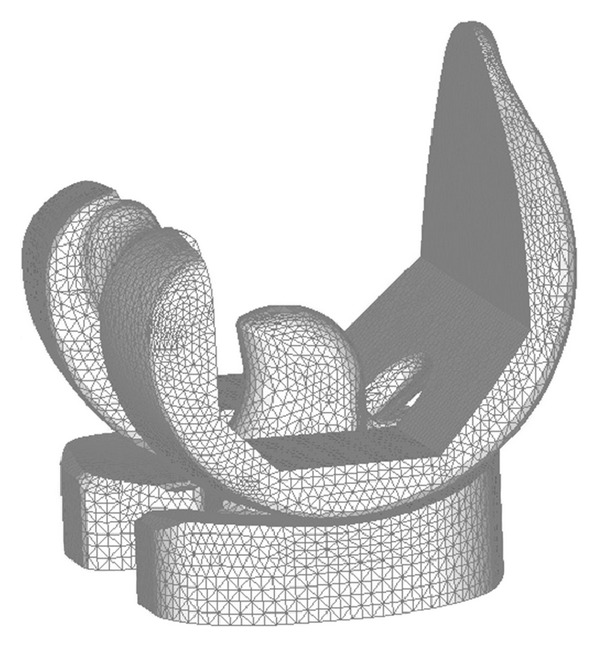
Mesh model of PS-type knee prosthesis for FEA analysis.

**Figure 3:**
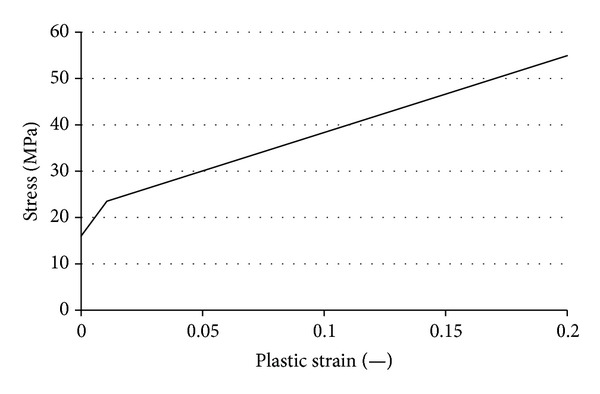
Bilinear relationship of stress-plastic strain curve of UHMWPE.

A posterior-stabilized total knee prosthesis, Scorpio NRG (Stryker Co., Kalamazoo, USA) was used for analysis (Figure 1). The feature of tibial insert design is symmetrical and flat, which enables flexible axial rotation. Three-dimensional FEA models, consisting of femoral component, tibial insert, and tibial component, were constructed from the CAD data obtained from the manufacturer, as illustrated in Figure 2. Tetrahedral meshes were generated on these models by FEMAP ver. 9.2 (Siemens PLM Software, Plano, USA). The number of nodes and elements was 28,254 and 121,536, respectively. The tibial insert made from ultra-high-molecular-weight polyethylene (UHMWPE) was assumed to be elastic-plastic material and to follow von Mises yield criterion. The nonlinear stress-strain relationship experimentally obtained was used (Table 1, Figure 3) [20].

Femoral and tibial components made of Co-Cr alloy were assumed to be rigid for reducing computational complexity. A coefficient of friction of articular surface was set to be 0.04. Four nonlinear springs were attached to tibial component in order to represent soft tissues around the knee. Its nonlinear force-displacement relation was given by [14]

(1) $$\begin{array}{c} F=\;0.18667d^{2}+\;1.3313d, \end{array}$$

where *F* and *d* are force and displacement under a knee with cruciate ligaments removed.

**Figure 4:**
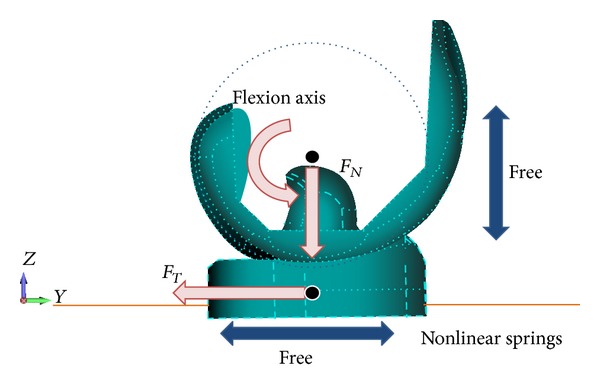
Boundary conditions of the current analysis. Femoral component is free along the vertical axis and rotates along the flexion axis. Tibial component is free along the AP axis and rotates along the vertical axis located at the center of component. The force *F*_*N*_ is applied to the femoral component and *F*_*T*_ is applied to the tibial component. Four linear springs, two in the front and two in the back, are attached to the tibial component in order to represent soft tissues around the knee.

**Figure 5:**
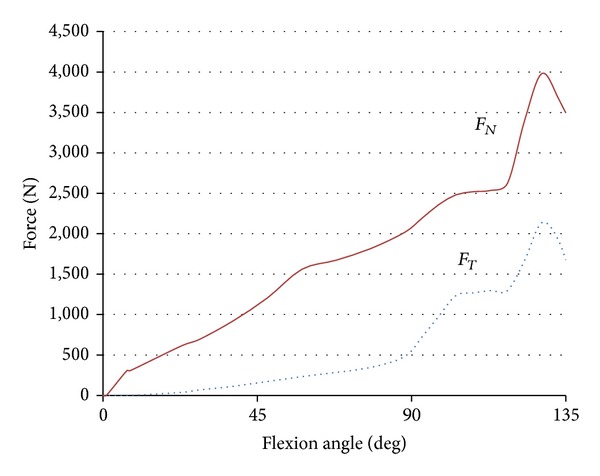
Force-flexion angle relationship of loading conditions. *F*_*N*_ is vertical load applied to the femoral component and *F*_*T*_ is horizontal load applied to the tibial component.

Boundary conditions are shown in Figure 4. The femoral component was allowed to translate in the vertical direction and rotate about a transverse axis to simulate flexion and extension. The tibial component was allowed to translate in the AP direction and rotate about a vertical axis located in the center of tibial condyles to simulate internal and external rotation [15]. Load conditions were referred to in the previous analysis [21]. Vertical load was applied to the femoral component which rotated from 0° to 135° of flexion while horizontal load along the AP direction was applied to the tibial component which internally rotated from 0° to 15° of rotation during knee flexion. The amount of vertical load increased gradually to its maximum level of 4000 N around 130° of flexion and the amount of AP direction load increased to 2100 N at the same angle (Figure 5). Three different initial conditions of tibial components, normal (NRM), internally rotated for 15° (IR), and externally rotated for 15° (ER), were analyzed. In the current analysis, the rotational alignment of femoral component was defined as a line through the center of both femoral fixation pegs. The rotational alignment of the tibial component was defined as a line along the posterior border. Two lines were configured as parallel in NRM position.

For FEA, explicit finite element codes LS-DYNA ver. 971 and LS-PREPOST ver. 4.0 (Livermore Software Technology Co., Livermore, USA) were utilized as a solver and a postprocessor. The maximum von Mises stress on post surface and condyle surface of tibial insert was analyzed separately.

## 3. Results

**Figure 6:**
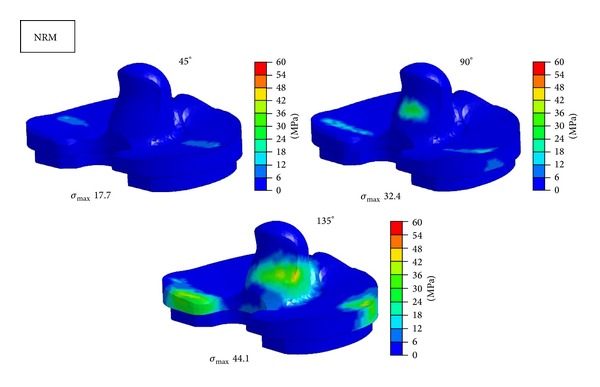
Maximum equivalent stress distribution in tibial insert at each position at the flexion angle of 45°, 90°, and 135°. *σ*_max⁡_ = maximum equivalent stress. NRM = normal.

**Figure 7:**
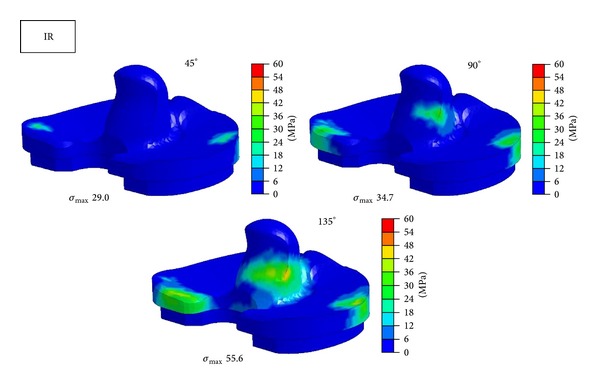
Maximum equivalent stress distribution in tibial insert at each position at the flexion angle of 45°, 90°, and 135°. *σ*_max⁡_ = maximum equivalent stress. IR = internal rotation.

**Figure 8:**
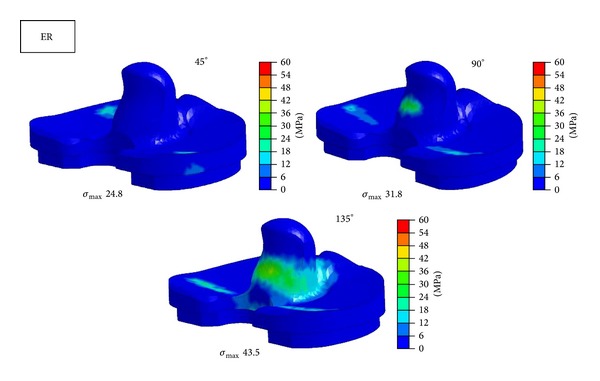
Maximum equivalent stress distribution in tibial insert at each position at the flexion angle of 45°, 90°, and 135°. *σ*_max⁡_ = maximum equivalent stress. ER = external rotation.

Figures 6, 7, and 8 show contours of maximum von Mises equivalent stress on tibial insert of NRM, IR, and ER positions at the flexion angle of 45°, 90°, and 135°. Concentrated stress on the edge of tibial insert was observed at 135° in NRM position and at 90° and 135° in IR malposition. ER malposition had no elevation of stress level on the edge.

**Figure 9:**
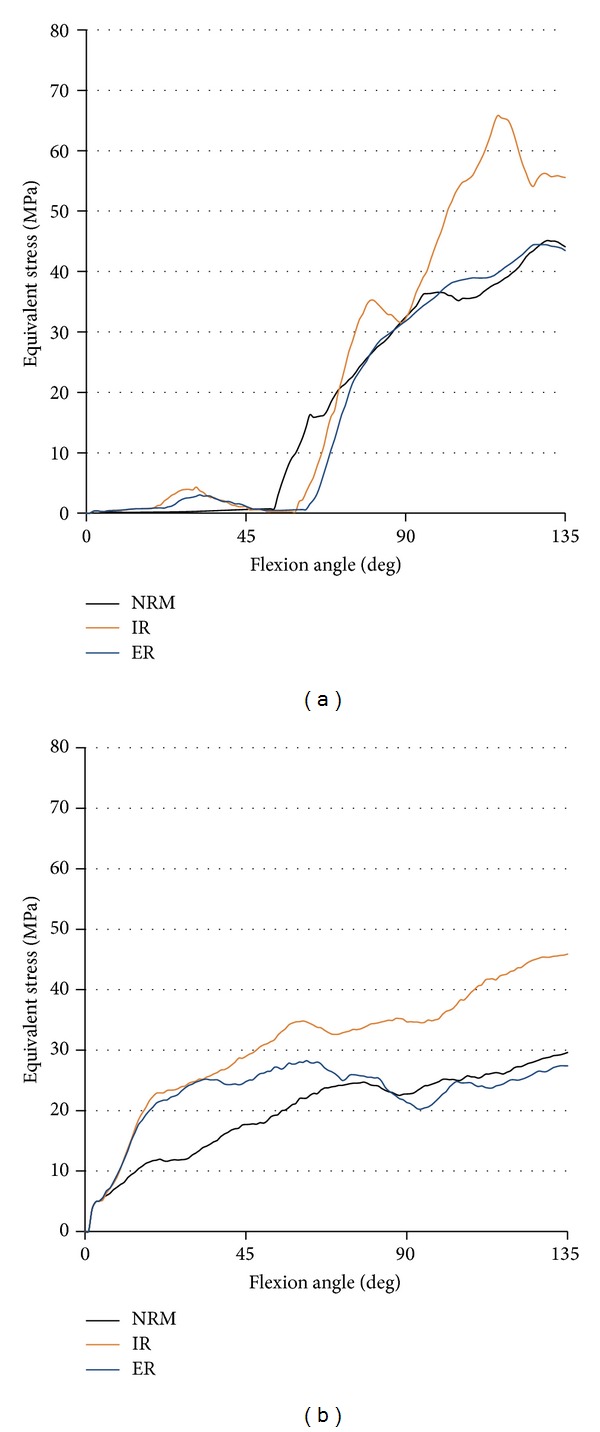
The equivalent stress (MPa) on tibial insert at each flexion angle. (a) Stress on post surface. (b) Stress on condyle surface.

The history of maximum Mises equivalent stress on the insert is shown in Figure 9. In each rotational position, the stress on post surface rapidly increased after the post-cam engagement which occurred at around 60° of knee flexion. Although ER malposition led almost to the same pattern of stress history as NRM position, IR malposition caused significant increase in the stress on post surface under high flexion (Figure 9(a)). The level of the stress of post surface in IR at 120° of flexion was 1.6 times as that in NRM. The stress on condyle surface was also high in IR malposition throughout knee flexion. ER malposition increased the stress on condyle surface under midflexion compared to NRM position (Figure 9(b)).

## 4. Discussion

Rotational alignment of femoral and tibial components affects the stress distribution on contact surface. Wear and fracture of polyethylene is a common complication of TKA. Biomechanical studies have demonstrated that the stress in polyethylene component is closely related to polyethylene failure [13]. There are many patterns in polyethylene damage, including crack, wear, and delamination [22, 23]. Sirimamilla et al. showed that peak stress caused crack propagation in cross-linked UHMWPE [24]. Accumulated crack could lead to delamination in the subsurface region. As the number of TKA performed is increasing and the procedure is performed on more culturally diverse populations, high flexion of the knee is in high demand. The result of the current study implies that malrotation of the tibial component increases the risk of early failure of the tibial polyethylene insert [14] and the impingement of components which could affect range of motion and lead to stiff knees [25]. Some of the revision surgeries are related to these complications. It is, therefore, important to analyze the effect of malposition on tibial inserts. Many researchers have investigated polyethylene wear using various analytical methods such as FEA and experimental measurement. Most studies, however, assumed normal position of each component [11]. A few studies, to our knowledge, have analyzed the effect of malrotation of tibial component using FEA.

Matsuda et al. investigated the contact stress on various types of tibial insert under 15° of malrotation of tibial component [8]. They used an electronic sensor to detect contact location in cadaver knees; then they measured peak and mean stresses on the insert under compressive load. Their results showed that the contact stresses were higher when tibial component was implanted in malrotation position. The stress levels were more than double compared to those of the neutral position. Our result was consistent with Matsuda's study.

Liau et al. investigated the effects of malalignment on stresses on tibial insert of total knee prostheses by calculating the contact stress and von Mises stress using FEA [13]. They constructed three different prostheses designs, the high conformity flat-on-flat design, high conformity curve-on-curve design, and medium conformity curve-on-curve design at a neutral alignment and three different malalignment models including the medial translation, internal rotation, and varus tilt of femoral component relative to the tibial component. Compression load was applied to the tibiofemoral joint at extended knee position. Their results showed that each malalignment model had higher stress on tibial insert and malrotation led relatively to lower stress on tibial insert than other malalignments. The maximum von Mises stress increased about 15% in internal malrotation. However, their analysis, like other studies using FEA, was under static condition. Our results revealed that internal rotation malrotation of tibial component for 15° increased the stress on tibial insert by more than 50% during high flexion of the knee.

Innocenti et al. investigated the sensitivity of patellofemoral and tibiofemoral contact forces to patient-related anatomical factors and component position in the different TKA prosthesis types including two types of fixed bearing prosthesis and two types of mobile bearing prosthesis [12]. Each prosthesis was virtually implanted on the cadaver leg model and it underwent a loaded squat between 0° and 120°. Their results showed that tibiofemoral contact forces are mostly affected by an anatomical location of medial collateral ligament. Their results showed that malrotation of tibial components also played a role in increasing tibiofemoral contact force by up to 16% in fixed bearing TKA models. The reason why this percentage is much lower than our result is that they set 5° of malrotation in their analysis. Moreover, different prosthesis design would have different pattern of stress distribution and stress level.

There are several limitations in the current study. First, every FEA has boundary conditions. Squatting is produced by muscle force and regulated by many soft tissues. In the current analysis, we applied vertical load to the femoral component and horizontal load on the tibial component and attached four springs to the tibial component so as to represent soft tissues around the knee joint. Although it is difficult to reproduce in vivo kinematics of the knee on computer simulation, parameters in the current study had been all experimentally examined and our model demonstrated the roll-back motion of the knee. Moreover, the stress level of our analysis was assumed to be consistent with previous study. Akasaki et al. investigated the contact stress on post surface using the same prosthesis as our analysis and the stress level at 90° of knee flexion was 35 MPa which was almost the same as our result [7]. Therefore, we consider our results as valid. Secondly, we did not consider the cyclic loading. We analyzed the stress on tibial insert during single squatting. Polyethylene wear was generated as a result of repeated loading on the surface. Finally, we analyzed one prosthesis which has relatively flat tibiofemoral joint. When we extend the current analysis to other prostheses such as more high conformity design prosthesis, the results would be different. Further investigation would be expected for future studies.

As the tibial insert of NRG is symmetrical and flat, the prosthesis is recognized to have a considerable extent of flexibility for axial malrotation. However, the results of this study revealed that excessive internal rotation malrotation increased the stress on tibial insert significantly. Therefore, internal rotation malrotation should be avoided when we use this prosthesis.

## 5. Conclusion

Although Stryker NRG has a flat design which produces flexible axial rotation, according to the current study, internal malrotation of tibial component caused a significant increase of stress level on tibial polyethylene insert. To reduce the risk of early failure of tibial insert, it is important to avoid internal malrotation of tibial component.

## Figures and Tables

**Table 1:** Material constants of UHMWPE tibial insert.

Density (kg/m^3^)	*E* (MPa)	*v* (—)	*σY* (MPa)
940	800	0.4	16
